# International Congress—Final Notice

**Published:** 1891-06

**Authors:** Pemberton Dudley

**Affiliations:** Gen. Secretary, A. I. H.; Fifteenth and Master Streets, Philadelphia, Pa.


### THE INTERNATIONAL CONGRESS—FINAL NOTICE.

The annual circular of the American Institute of Homœopathy will have reached the profession before this notice appears in print. If any homœopathic physician has failed to receive a copy, the undersigned will mail one on application.

There is not a single indication pointing to a failure of the Convention. The fear that it might be international only in name has no longer any warrant in fact. There will be representatives present from England, France, Germany, Russia, and probably some other European countries, and of our trans-atlantic brethren there will be at least twenty-five of them represented either by essays or reports, or by their personal presence.

A casual examination of the list of papers and addresses to be presented will show that the Convention is not likely to follow, altogether, the well-beaten track of the typical society meeting. In its effort to secure the discussion of broad and comprehensive questions and issues, the Convention has not labored in vain. The profession has approved and supported the effort.

It is requested that the instructions for securing reduced rates on railroads shall be read with great care. Every direction necessary will be found there. Also that physicians not members of the Institute act promptly on the suggestions about uniting with that body. And also that each of those who attend shall, before leaving home, decide which of the essays he or she can discuss to the greatest advantage of the profession and come prepared to do so.

PEMBERTON DUDLEY, M. D.,
*Gen. Secretary, A. I. H.*

FIFTEENTH AND MASTER STREETS,
PHILADELPHIA, PA., May 18th, 1891.

